# Novel Hydrogel-Mediated Lentiviral Gene Delivery via Intravesical Administration for Bladder Cancer Treatment

**DOI:** 10.3390/pharmaceutics17020143

**Published:** 2025-01-21

**Authors:** Ching-Wen Liu, Po-Hen Chen, Kai-Jen Lin, Yu-Ting Cheng, Li-Ching Chang

**Affiliations:** 1 Department of Natural Biotechnology, Nanhua University, No. 55-Xue Hai Tang, Sec. 1, Nanhua Rd., Dalin Township, Chiayi County 622, Taiwan; fruit02270428@gmail.com (C.-W.L.); ytcheng530@gmail.com (Y.-T.C.); 2 Department of Medical Research, E-Da Hospital, Kaohsiung 824, Taiwan; pohan1123@gmail.com; 3 Department of Pathology, E-Da Hospital, No. 1, Yida Road, Yanchao District, Kaohsiung 824, Taiwan; ed102328@edah.org.tw; 4 School of Medicine, I-Shou University, No. 8, Yida Road, Yanchao District, Kaohsiung 824, Taiwan; 5 Department of Pharmacy, E-Da Hospital, I-Shou University, No. 1, Yida Road, Yanchao District, Kaohsiung 824, Taiwan

**Keywords:** urothelial bladder cancer, WW domain-containing oxidoreductase (WWOX), reactive oxygen species (ROS)

## Abstract

**Background/Objectives**: Bladder urothelial carcinoma is a frequent malignant tumor of the urinary system, characterized by its high rates of recurrence and resistance to chemotherapy. This study explored the beneficial effects of overexpressing WW domain-containing oxidoreductase (WWOX) in AY-27 cells encapsulated in an injectable gelatin hydrogel for potential therapeutic applications in bladder cancer. **Methods**: AY-27 cells were genetically transduced with lentiviruses (LV) to overexpress WWOX (LV-WWOX) and subsequently encapsulated in a gelatin hydrogel. The mechanical properties and morphology of the hydrogels were assessed using transmission electron microscopy (TEM) and scanning electron microscopy (SEM). The therapeutic efficacy of this approach was evaluated using an F344/AY-27 rat orthotopic bladder cancer model, in which the LV-WWOX-hydrogel (H-LV-WWOX) was administered via intravesical instillation. **Results**: The gelatin hydrogel formulation demonstrated excellent biocompatibility, stability, and controlled release. In a rat orthotopic model, intravesical instillation of H-LV-WWOX significantly enhanced local immune responses, resulting in notable tumor regression. Compared to the sham-treated group, this approach reduced systemic toxicity and improved overall treatment outcomes. The anticancer effect of WWOX can be attributed to its ability to amplify TNF-α-induced reactive oxygen species (ROS) generation. This ROS-mediated pathway leads to enhanced apoptosis and DNA damage in cancer cells, highlighting the potential mechanism through which *WWOX* exhibits tumor-suppressive activities. **Conclusions**: These findings support the therapeutic potential of WWOX overexpression in gelatin hydrogels for bladder cancer treatment and warrant further clinical investigation.

## 1. Introduction

WW domain-containing oxidoreductase (*WWOX*) is a tumor suppressor gene located at a common fragile site [1]. Numerous studies have shown that WWOX expression is often lost or reduced in various cancers, including bladder, breast, liver, and nasopharyngeal cancers [2,3,4]. WWOX plays a role in various signaling networks that control cell growth, metabolic processes, and programmed cell death. As a tumor suppressor, WWOX acts as a downstream effector of the tumor necrosis factor (TNF) signaling pathway [5]. Consequently, both WWOX and NF-κB can be activated during TNF-signaling or in response to stressful conditions. Emerging evidence suggests that WWOX modulates caspase activity, thereby influencing the apoptotic response [6]. This interaction underscores the potential of WWOX as a therapeutic target in cancer treatment, particularly in strategies aimed at restoring apoptosis in tumor cells [7]. Impairment of caspase function has been associated with various disorders, including cancer, characterized by decreased apoptosis and excessive cellular proliferation. Caspases also play roles in necrosis and inflammation [8]. These enzymes contain a cysteine residue in their active site, and upon activation, cleave target proteins at specific aspartic acid residues to induce programmed cell death [9].

Gelatin is a natural protein-derived material that is often used in the creation of medical hydrogels due to its low immunogenic response, ability to enhance cell adhesion, and excellent biocompatibility [10]. Recently, functional thermosensitive gelatin hydrogels have emerged as promising therapeutic agents for biomedical applications [11]. These hydrogels swell until the thermodynamic forces driving the swelling are balanced by the elastic and retractive strengths of the crosslinks. Hydrogels formed through macromolecular crosslinking motifs are particularly appealing for use in biomaterials and drug delivery systems [12].

Gene therapy using hydrogel platforms has the capacity to greatly advance the field compared with other strategies [13]. By entrapping vectors within hydrogels, these platforms enable sustained release, maintain elevated local vector concentrations, and enhance the likelihood of cellular internalization [14]. Thermosensitive scaffolds are natural or synthetic materials that undergo a solution-to-gel transition at 37 °C, the body’s temperature. The overall efficiency of gene delivery depends on how the hydrogel influences both cell infiltration and the rate of vector release because these factors collectively determine the success of the gene delivery process [15].

Systemic therapy is effective for targeting metastasis but is also associated with systemic toxicity and potential immunogenicity due to accumulation in major organs. In contrast, hydrogel-mediated delivery of gene therapeutics for cancer therapy enables the prolonged retention of nanovectors around the tumor, enhancing their uptake by targeted cancer cells [16]. Hydrogels have the potential to facilitate long-term, sustained local delivery of gene therapy, which may reduce adverse effects and enhance the efficacy of targeting primary tumors. By providing a controlled release environment, hydrogels ensure that therapeutic agents are delivered directly to the tumor site over an extended period, potentially improving therapeutic outcomes while minimizing systemic exposure and toxicity [17].

This study introduced a gelatin hydrogel with electrostatic affinity for LV and the ability to efficiently deliver them into bladder cancer cells. An in vivo study using the AY-27/F-344 rat model was designed to evaluate the hydrogel system’s antitumor potential and assess signs of toxicity. This study explored the potential of H-LV-WWOX for intravesical instillation in bladder cancer. The primary goal is to enhance localized therapeutic efficacy by leveraging the synergistic effects of immunomodulatory agents within a controlled delivery matrix. By optimizing the formulation and administration of this hydrogel system, we aimed to reduce tumor recurrence and improve the overall outcomes of bladder cancer treatment.

## 2. Materials and Methods

### 2.1. Materials

Gelatin, pronase, and glutaraldehyde were obtained from Sigma-Aldrich (St. Louis, MO, USA). All cell culture media and related reagents were sourced from Gibco BRL (Grand Island, NY, USA). The concentrations of viral particles were measured using an HIV-1 p24 Antigen ELISA kit (ZeptoMetrix, Franklin, MA, USA). In situ urothelial carcinoma was established by intravesical instillation of AY-27 cells into the bladders of Fischer F344 rats, which were purchased from the National Laboratory Animal Center (NLAC) in Taipei, Taiwan. All in vivo studies involving rats were conducted in compliance with the guidelines of the Association for Assessment and Accreditation of Laboratory Animal Care and were approved by the I-Shou University Institutional Animal Care and Use Committee (IACUC) under approval number IACUC-ISU-102025.

### 2.2. Characterization of Hydrogels

The hydrogel was prepared using A-type gelatin (Sigma-Aldrich, St. Louis, MO, USA) with 175 bloom numbers, following the methods described previously [18]. The Fourier transform infrared (FTIR) spectra of the hydrogel samples were obtained using the thin transparent tablet technique with potassium bromide. KBr and the samples were prepared by grinding them into a powder using an amalgamator (WIG-L-BVG, 31210-3A, USA), mixed, and pressed under a pressure of 200 MPa to form vacuum tablets. Particle size distribution and mean diameter were determined using an N5 Submicron Particle Size Analyzer (Beckman, Hialeah, FL, USA). FTIR spectra were recorded in the wave number range from 4000 to 400 cm^−1^ on a Bomem Hartmann & Braun MB-100 series FTIR spectrophotometer. The surface morphology of the freeze-dried hydrogels was analyzed using SEM (JEOL Ltd., Akishima, Tokyo, Japan). ImageJ software was used to quantify and analyze the pore size of each hydrogel group. The morphologies of the hydrogels were observed using a JEM-1230 (JEOL, Japan) TEM at an accelerating voltage of 80 kV. To preserve their morphology, the hydrogels were frozen in liquid nitrogen before being freeze-dried. The sectioned gels were then mounted on metal holders and vacuum-coated with a gold layer for SEM examination.

### 2.3. Viral Assembly and Incorporation with LV-WWOX

The LV was prepared for the studies using established methods. The lentiviral packaging vectors (pMDL-GagPol, pRSV-Rev, and pIVS-VSV-G) were co-transfected with pLKO_AS2.puro-WWOX (LV-WWOX) or pLKO_AS2.puro (LV) into 293T cells using TranslT-LT1 (Mirus Bio, Madison, WI, USA). LV production was carried out in 293T cells, which were cultured in Dulbecco’s modified Eagle’s medium (DMEM) containing 10% fetal bovine serum at 37 °C with 5% CO_2_. After 48 h, the supernatant was collected and filtered through a 0.45-micron filter. The infectious titer of LV-WWOX was determined by counting the number of cells expressing WWOX two days after incubating serially diluted viruses with 293T cells. H-LV-WWOX was applied to AY-27 cells for 18 h at a multiplicity of infection (MOI) of 9.

### 2.4. Cytotoxic Assay

The AY-27 rat bladder cancer cell line (kindly provided by Professor R. Moore, University of Alberta, Edmonton, AB, Canada) was cultured in RPMI-1640 medium containing 10% fetal bovine serum (FBS) and 2% L-glutamine at 37 °C in a 5% CO_2_ environment. Cell viability was assessed using the CellTiter 96 Aqueous Non-Radioactive Cell Proliferation Assay following the manufacturer’s protocol (Promega, Madison, WI, USA). A total of 1 × 10^4^ AY-27 cells were seeded in 96-well plates and treated with LV, LV-WWOX, H-LV, and H-LV-WWOX for 18 h. The results are expressed as the mean ± SEM from four independent experiments.

### 2.5. Immunocytochemistry and Western Blotting

The cells were permeabilized using 0.2% Triton X-100 in PBS. After blocking with antibody diluent reagent (Dako, Carpinteria, CA, USA), the cells were incubated with primary antibodies targeting WWOX (Imgenex, San Diego, CA, USA) and TNF-α (BioLegend, San Diego, CA, USA) in antibody diluent reagent (Dako, Carpinteria, CA, USA) for 1 h at room temperature (RT). The secondary antibody was applied in an antibody diluent reagent (DAKO, Glostrup, Denmark) for 1 h at RT. The stained cells were mounted with Shield Mounting Medium (DAKO, Glostrup, Denmark) and photographed using a Nikon Eclipse Ti inverted microscope. AY-27 cells were washed twice with cold PBS and lysed in TBS containing 1 mM DTT, 1 mM EDTA, 0.1% SDS, 0.2% Triton X-100, and complete protease inhibitor mixture (Roche, Indianapolis, IN, USA). Proteins were separated by SDS-PAGE and transferred onto polyvinylidene difluoride (PVDF) membranes (Boehringer Mannheim, Indianapolis, IN, USA). Membranes were probed with antibodies specific for actin (Merck Millipore, Darmstadt, Germany), WWOX (Imgenex, San Diego, CA, USA), caspase-3 (Cell Signaling, Danvers, MA, USA), and TNF-α (Cell Signaling, Danvers, MA, USA), and incubated with HRP-conjugated anti-mouse IgG or anti-rabbit IgG antibodies. Signals were detected using the ECL kit (Merck Millipore, Darmstadt, Germany) and visualized by autoradiography.

### 2.6. Ethics Statement and Cystometrogram (CMG) and Data Analysis

All in-life rat studies were performed in accordance with the guidelines approved by the Association for Assessment and Accreditation of Laboratory Animal Care and authorized by the I-Shou University Institutional Animal Care and Use Committee (approval number IACUC-ISU-102025).

CMGs were performed as described previously [18]. Fischer F344 rats (8 weeks old) were anesthetized using Zoletil-50 (1 mg/kg). Prior to each CMG, the bladder was emptied, and a urethral catheter was placed to fill the bladder and measure bladder pressure. The catheter was connected via a T-tube to a syringe pump (KDS250, KD Scientific Corp., MA, USA), pressure transducer, and amplifier (ML866 and ML224, PowerLab, AD Instruments, Springs, CO, USA). Data were captured on a chart recorder and converted into digital format for computer analysis (LabChart 7; ADInstruments, Windows 7 system). The bladder was subsequently infused with 500 μL of normal saline (as a control) and LV-WWOX-loaded hydrogels at a constant rate of 0.07 mL/min, while pressure was continuously monitored through the catheter.

Voiding contractions were characterized by an increase in bladder pressure leading to urine expulsion. CMG recordings were taken until bladder pressure stabilized, with a minimum of five filling/voiding cycles measured in each rat before drug administration. These cycles served as the baseline values. The CMG parameters recorded for each animal included duration of non-voiding contractions (without urine leakage during bladder infusion) and bladder compliance. Bladder compliance was calculated as infused volume (µL) divided by threshold pressure (ΔcmH_2_O).

### 2.7. In Vivo Urothelium Permeability and Histologic Analysis

F344 rats were anesthetized, and a urethral catheter was inserted to catheterize the bladder. In situ urothelial cancer was then induced through intravesical instillation of AY-27 rat bladder tumor cells. To facilitate tumor seeding, the bladder mucosa was conditioned with 0.4 mL of 0.1 N potassium hydroxide (KOH) for 15 s, followed by neutralization with 0.4 mL of 0.1 N hydrochloric acid (HCl) for 15 s. The bladders were then drained and flushed with sterile normal saline. Immediately after bladder conditioning, AY-27 cells (1 × 10 units) were instilled and left indwelling for at least 1 h. The rats were rotated by 90° every 15 min to ensure that the entire bladder was exposed to the tumor cell suspension. One hour later, the catheter was removed, and the rats were permitted to void spontaneously.

On day 1 after tumor implantation, F344 rat bladders were intravesically instilled with LV or LV-WWOX on days 8, 10, and 12. All rats were sacrificed on day 14. On day 14, the bladders were removed, fixed in 4% formalin overnight, dehydrated, and embedded in paraffin. The paraffin-embedded tissues were subsequently sectioned into 4 μm slices and stained with hematoxylin and eosin (H&E) for histological examination.

### 2.8. Measurement of Intracellular ROS

To evaluate ROS production in sham-treated tumors and the groups LV, LV-WWOX, H, H-LV, and H-LV-WWOX, dihydroethidium (DHE) staining was performed following the manufacturer’s instructions, as previously described. Briefly, the primary organs were pretreated with the specified interventions and subsequently incubated with DHE (10 μM; Sigma, St. Louis, MO, USA) for 30 min in the dark. Fluorescence microscopy was used to observe the samples at 400× magnification (Eclipse 80i, Nikon, Tokyo, Japan), and the positive staining areas were quantified using ImageJ software (version 1.42).

### 2.9. Statistical Analysis

For data that exhibited equal variance, statistical analysis was performed using analysis of variance (one-way ANOVA) followed by Tukey’s post hoc test, with significance set at *p* < 0.05. All group data are presented as mean ± SEM. Statistical analysis was performed using Student’s *t*-test and one-way analysis of variance (ANOVA). Statistical significance was set at a *p*-value of <0.05.

## 3. Results

### 3.1. Comprehensive Physicochemical and Morphological Characterization of Lentivirus Hydrogel

**Figure 1:**
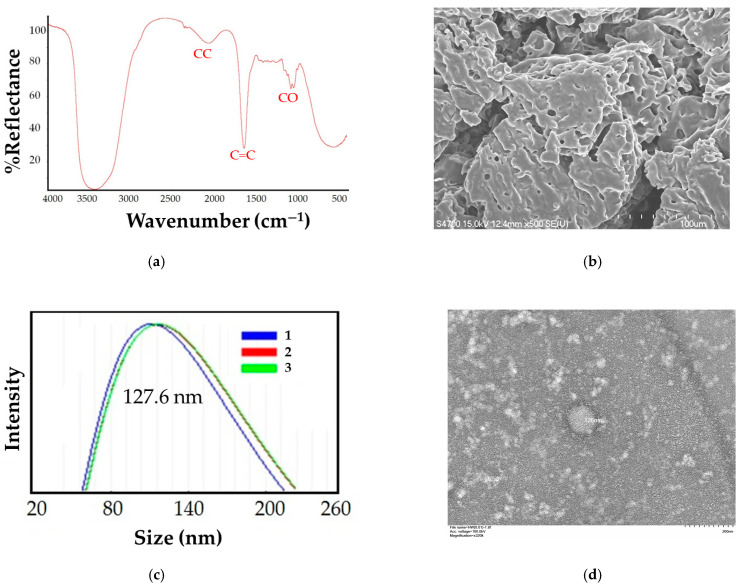
Characterization of hydrogels. (**a**) FTIR spectrum hydrogels and (**b**) FE-SEM picture of lentivirus coupled to hydrogel (magnification, ×500). (**c**) Particle size distribution. (**d**) TEM picture of lentivirus coupled to hydrogel (magnification, ×320,000).

To examine the microstructure of the lyophilized hydrogel, we conducted a physicochemical analysis focusing on pore size, FTIR, and SEM imaging. FTIR analysis demonstrated the specific chemical interactions between the active components of the composite hydrogel system. The FTIR spectrum of the hydrogel samples (Figure 1a) showed a carbonyl stretch in the gelatin at 1620–1680 cm^−1^, along with additional bands between 1200 and 1000 cm^−1^ corresponding to C–O stretches. The presence of these polarized functional groups suggests significant intermolecular chemical interactions within the hydrogel, which likely contribute to its stability and functionality. FE-SEM images at ×500 magnification revealed the cross-sectional surface of the hydrogels. The pore size of the hydrogels, measured using ImageJ software, was found to be 4.5 ± 0.3 μm. Figure 1b shows the microstructure of the hydrogel, which has a connected porous structure with uniform pores that facilitate water absorption and swelling. The formation of these pores is strongly influenced by ice crystal formation and varying the crosslinking levels likely leads to different pore sizes. Pore size is a critical factor influencing hydrogel performance because high porosity and interconnected pores are essential for promoting cellular activity, including cell penetration, nutrient transport, and an optimal degradation profile.

The HLV particle size analysis, which revealed an average particle size of approximately 127.6 ± 2.8 nm (Figure 1c). When the hydrogel was loaded with the lentivirus, TEM imaging indicated that the lentivirus was partially dispersed within the hydrogel matrix. TEM further confirmed that the hydrogel particles exhibited a spherical core–shell structure, with an average size of approximately 121.7 ± 10 nm (Figure 1d). The close agreement between these measurements suggests that the lentivirus-loaded hydrogel particles had a consistent and uniform size, indicating reliable and reproducible particle formation.

### 3.2. LV-WWOX-Hydrogel Vector Release and Cell Transduction Efficiency

**Figure 2:**
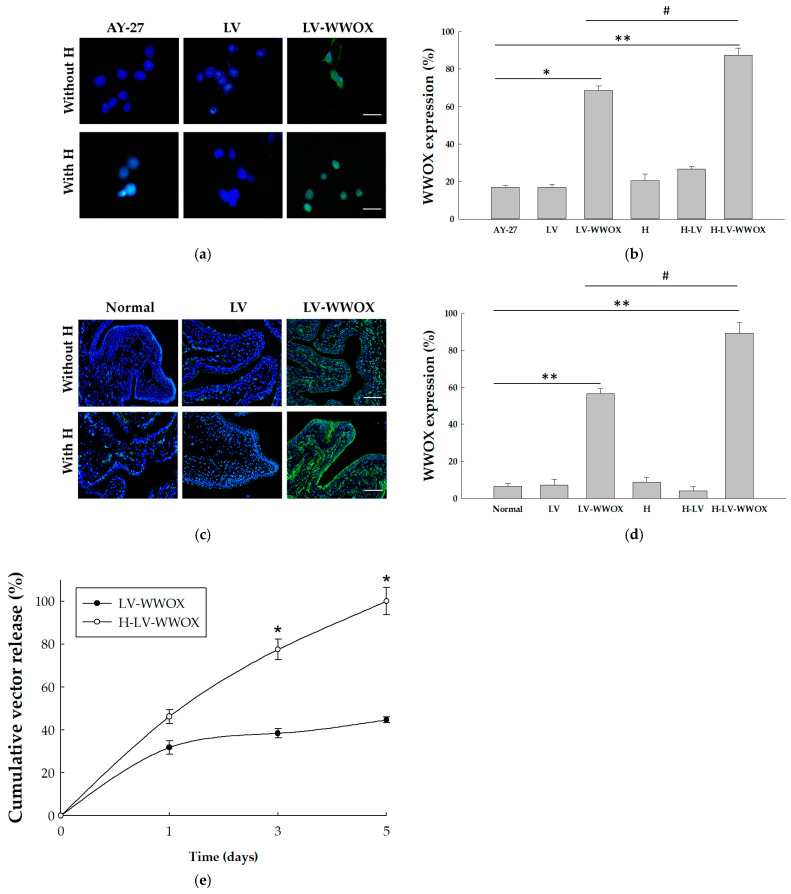
The effect of overexpressed WWOX: (**a**) Localization of WWOX in WWOX-overexpressed AY-27 cells, as determined by immunofluorescence analysis (magnification: ×200). (**b**) Quantification of WWOX expression levels in positive cells (%). * *p* < 0.05 and ** *p* < 0.01 compared to the AY-27 group; # *p* < 0.05 compared to the LV-WWOX group. (**c**) Representative images of tissue samples stained with DAPI (nuclei) at week 1 following in vivo intravesical instillation and implantation of virus-loaded hydrogel (magnification of ×200). (**d**) Quantification of WWOX expression levels in positive cells (%). ** *p* < 0.01 compared to the normal control group; # *p* < 0.05 compared to the LV-WWOX group. (**e**) The cumulative release of lentivirus from hydrogels (H-LV-WWOX) or LV-WWOX alone in 293T cells was evaluated by measuring HIV-p24 content (*N* = 3). The data are presented as mean ± SEM. * *p* < 0.05 indicates a significant difference between LV-WWOX and H-LV-WWOX.

The interaction between the LV-WWOX-hydrogel (H-LV-WWOX) and cells, as well as its internalization, transduction efficiency, and gene expression, requires further investigation. To identify the optimal H-LV-WWOX, LV and hydrogel complexes were prepared by incubating them in medium at room temperature. The cell transduction efficiency was assessed by evaluating WWOX expression. WWOX expression in lentivirus-infected cells was measured 48 h post-transfection using immunofluorescence staining (Figure 2a). As shown in Figure 2b, WWOX protein expression levels in AY-27 cells infected with LV-WWOX were 4.0-fold (*p* < 0.05) higher compared to control AY-27 cells. In AY-27 cells infected with H-LV-WWOX, WWOX protein expression levels were 5.1-fold (*p* < 0.01) higher compared to control cells. Furthermore, H-LV-WWOX achieved the highest transduction efficiency (87.4%), compared to only 68.6% for LV-WWOX (*p* < 0.05). Using one-way ANOVA, the F-test results indicated a significant difference among the six groups, with a *p*-value < 0.05. Next, we assessed the gene delivery efficiency of LVs encapsulated in gelatin hydrogels into the rat urothelium through intravesical instillation. Seven days after LV-WWOX instillation, WWOX expression was localized to the top one or two layers of the umbrella cells in the urothelium (Figure 2c). However, following H-LV-WWOX treatment, WWOX expression was detected in all layers of the urothelium and extended into the subepithelial connective tissue. In contrast, WWOX expression was not detected in the control urothelium, hydrogel, LV, or H-LV alone. Cell nuclei were counterstained with DAPI (blue). These data indicate that hydrogels improve LV delivery to the urothelium through intravesical instillation into the rat bladder in vivo. As shown in Figure 2d, WWOX protein expression levels in the umbrella cells infected with LV-WWOX were 1.58-fold (*p* < 0.05) higher than those in the H-LV-WWOX group. Additionally, WWOX protein expression levels in the umbrella cells with LV-WWOX were 8.6-fold (*p* < 0.05) higher than those in the normal control, while levels in the umbrella cells with H-LV-WWOX were 13.5-fold (*p* < 0.01) higher than those in the normal control. One-way ANOVA was performed, and the F-test results revealed a significant difference among the six groups, with a *p*-value < 0.05.

Next, the release of LV from the hydrogel-loaded scaffolds was investigated, as it influences the availability of the vector to the infiltrating cells. The capacity of the hydrogels to function as scaffolds for LV release was evaluated by measuring HIV-p24 content, which acts as an indicator of lentiviral titers. LV-WWOX alone and hydrogels containing H-LV-WWOX were infected into AY-27 cells. As shown in Figure 2e, H-LV-WWOX produced a significantly greater cumulative and long-lasting release of the p24 capsid protein compared to LV-WWOX alone. The cumulative release of LV-WWOX/H-LV-WWOX was 22.7% and 21% initially (0.9-fold), 42.9% and 57.5% on day 1 (1.3-fold), 47.9% and 82.3% on day 3 (1.7-fold, *p* < 0.05), and 52.6% and 100% on day 5 (1.9-fold, *p* < 0.01), respectively. This cumulative release profile confirmed the potential of hydrogels as effective scaffolds for the sustained release of LV-WWOX.

### 3.3. Bladder Function in F344 Rat

**Figure 3:**
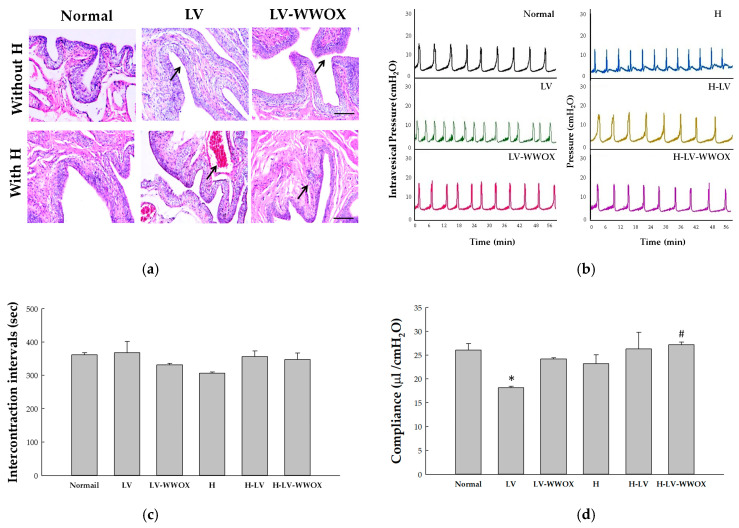
Analysis of bladder function in F344 rats was subjected to intravesical instillation of hydrogels containing lentivirus. (**a**) Pathohistological examination: F344 rats were subjected to intravesical instillation with Normal (N), LV, LV-WWOX, H, H-LV, and H-LV-WWOX treatments. The bladders were then stained with hematoxylin and eosin, and images were captured at a magnification of ×200. (**b**) Representative continuous cystometrogram recordings: Continuous CMG recordings were obtained from F344 rats with intravesical infusion of saline (0.7 mL/min) under anesthesia. (**c**) Group-wise analysis of the intercontraction interval (seconds) and (**d**) bladder compliance (μL/cmH_2_O) was conducted across the groups. Significant differences were observed with * *p* < 0.05 compared to the normal group; # *p* < 0.05 when compared to the LV-WWOX group.

The use of lentiviral vectors in gene therapy offers significant promise but also raises safety concerns, particularly regarding the potential for severe immune reactions. To address these concerns, the safety profile of H-LV-WWOX was evaluated by assessing its effects following intravesical instillation in a rat model, with a focus on bladder inflammation and tissue damage. LVs, LV-WWOX, and their respective hydrogel formulations (H-LV and H-LV-WWOX) were introduced into the rat urothelium via intravesical instillation. This approach enabled us to assess the localized impact of these treatments on bladder tissue. Histological studies were conducted on specimens from the control, LV, LV-WWOX, H, H-LV, and H-LV-WWOX groups after 60 d (Figure 3a). Treatment with LV or LV-WWOX alone resulted in mild thickening of the urothelium (indicated by arrows), with no significant tissue damage or hemorrhage. These findings suggest a generally safe profile with a minimal inflammatory response. In contrast, the H-LV and H-LV-WWOX groups exhibited mild inflammation and hemorrhage, indicating that, although the hydrogel delivery system was effective, it may contribute to localized tissue irritation. According to the hematology data, all values are within the normal range for rats, including white blood cell count (6.0–18.0 × 10^9^/L) and lymphocyte percentage (normal range: 60–90%). The white blood cell count observed after 2 months in the Normal, H-LV, and H-LV-WWOX treatment groups was 9.0 ± 3.1 × 10^9^/L, 8.0 ± 1.3 × 10^9^/L, and 6.0 ± 2.9 × 10^9^/L, respectively. The lymphocyte percentage observed after 2 months in the Normal, H-LV, and H-LV-WWOX treatment groups was 84.0 ± 3.0%, 79.5 ± 1.5%, and 78.5 ± 1.5%, respectively.

To further evaluate the safety and potential inflammatory responses associated with H-LV-WWOX, the impact of transurethral open cystometry (CMG) under saline infusion was also assessed. This assessment was designed to determine whether the procedure itself or the presence of H-LV-WWOX affected bladder function. Transurethral open CMG with saline infusion is considered non-inflammatory and should not activate inflammatory pathways or induce LV-WWOX expression. Representative images from each treatment group (LV, LV-WWOX, H-LV, H-LV-WWOX, and control) are shown in Figure 3b.

Bladder capacity was assessed by measuring the volume of normal saline infused into the bladder until micturition occurred. The intercontraction interval (ICI; s) is defined as the time elapsed between two successive voiding cycles. The ICI observed in the Normal, LV, LV-WWOX, H, H-LV, and H-LV-WWOX treatment groups was 361.0 ± 7.0, 368.0 ± 33.2, 331.0 ± 5.0, 356.5 ± 16.8, and 347.0 ± 20.3 s, respectively. Analysis of the ICI data revealed no significant differences (*N* = 4; *p* > 0.05; Figure 3c). Bladder compliance, calculated as the infused volume (μL) divided by threshold pressure (ΔcmH_2_O), was higher in the H-LV-WWOX group compared to the LV-WWOX group (27.2 ± 0.5 vs. 24.2 ± 0.2, *p* < 0.01). The cystometrogram results indicated that the mucoadhesive H-LV-WWOX significantly increased bladder compliance (Figure 3d). Notably, bladder compliance in the LV group was lower. Overall, this study successfully demonstrated that the mucoadhesive hydrogel functions as an effective intravesical gene delivery scaffold without impacting voiding cycles.

### 3.4. Inhibition of AY-27 Cell Proliferation and Induction of Apoptosis upon Treatment with LV-WWOX-Incorporated Hydrogels

**Figure 4:**
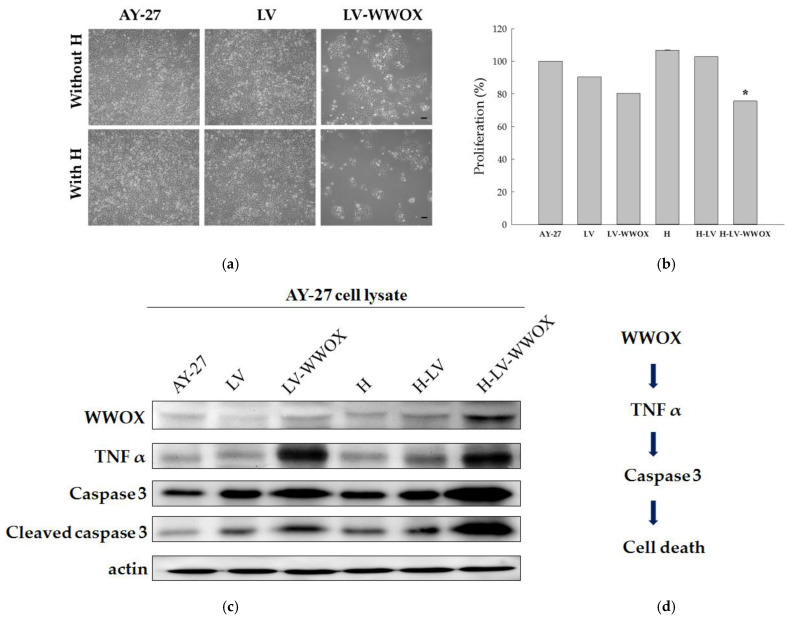
Inhibit cell proliferation and caspase-3 activation in AY-27 cells through H-LV-WWOX. (**a**) Cell morphological and (**b**) proliferation (24 h) changes after being treated with LV, LV-WWOX, H, H-LV and H-LV-WWOX. The AY-27 cells were examined with a microscope at ×100 magnification. Significant differences were observed with * *p* < 0.05 compared to the normal group. (**c**) AY-27 cells were treated with LV, LV-WWOX, H, H-LV, and H-LV-WWOX, respectively. The expression levels of WWOX, caspase-3, cleaved caspase-3, and TNF-α were determined by western blotting. Actin was used as the loading control. (**d**) Model for the role of WWOX in TNF-α-mediated apoptosis.

This study aimed to explore the potential of combining LVs with hydrogels to enhance their interactions with cancer cells. This combination improves the delivery and efficacy of therapeutic hydrogels. Treatment with H-LV-WWOX induced noticeable morphological changes in the AY-27 cells (Figure 4a). Viral–hydrogel complexes (LV-hydrogel), consisting of 10^5^ TU of LV-WWOX coupled with the hydrogel, were incubated with AY-27 cells for 24 h, and the interactions were assessed using the MTS assay. As shown in Figure 4b, hydrogels exhibited cell viability greater than 100%, indicating that the hydrogels significantly promote cell proliferation. The viability of AY-27 cells after treatment with LV, LV-WWOX, H, H-LV, and H-LV-WWOX was 90.5 ± 0.0%, 80.3 ± 0.1%, 106.8 ± 0.1%, 102.8 ± 0.0%, and 75.7 ± 0.0%, respectively.

To investigate the mechanism underlying H-LV-WWOX-induced apoptosis, the intracellular activity of caspase-3, a key enzyme in the apoptotic pathway, was assessed. Following treatment with LV-WWOX or H-LV-WWOX, AY-27 cells showed increased caspase-3 activity, particularly in the H-LV-WWOX group (Figure 4c). As shown in Figure 4c, the expression levels of caspase-3 protein in AY-27 cells infected with H-LV-WWOX were 1.56-fold higher (*p* < 0.01) compared to those infected with LV-WWOX. Additionally, the cleavage of caspase-3 protein expression levels in AY-27 cells infected with H-LV-WWOX was 7.78-fold higher (*p* < 0.001) than in the LV-WWOX group. The WWOX protein expression levels in the AY-27 cells infected with H-LV-WWOX were 1.70-fold higher (*p* < 0.005) compared to those infected with LV-WWOX. Furthermore, TNF-α protein expression levels in LV-WWOX-infected AY-27 cells were 2.67-fold higher (*p* < 0.05) than in the control group. Finally, TNF-α protein expression levels in H-LV-WWOX-infected AY-27 cells were 2.74-fold higher (*p* < 0.01) than in the control group. Untreated cells and those treated with LV alone exhibited minimal caspase-3 expression, underscoring the specificity of WWOX in driving apoptosis. These findings suggest that modulating WWOX levels can influence the phenotypes induced by low TNF-α expression. Specifically, changes in WWOX expression correlated with variations in caspase-3 activity, indicating that WWOX enhances the apoptotic response to TNF-α by promoting caspase-3 activation and facilitating cell death. A potential mechanism for TNF-α’s role in this WWOX-mediated cell death pathway is its regulation of reactive oxygen species (ROS) (Figure 4d).

### 3.5. In Vivo AntiTumor Effect of H-LV-WWOX

**Figure 5:**
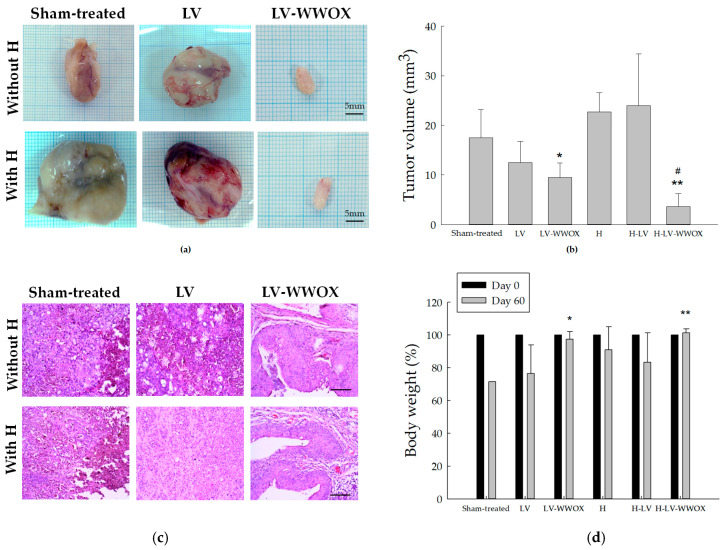
Effects of Intravesical Instillation of H-LV-WWOX on Tumor Regression. (**a**) Images of excised tumors on day 60. (**b**) The tumor volume showed significant differences, with * *p* < 0.05, ** *p* < 0.01 compared to the sham-treated tumors and # *p* < 0.05 for LV-WWOX versus H-LV-WWOX. (**c**) Histological findings of the primary organs are shown, with a scale bar of 100 μm (magnification of ×200). (**d**) Comparative body weight change (%) between day 0 and day 60 of the experiment in the control and experimental groups. * *p* < 0.05 for sham-treated tumors versus LV-WWOX; ** *p* < 0.01 for sham-treated tumors versus H-LV-WWOX.

To further assess the anticancer efficacy of WWOX, we established an F344/AY-27 rat orthotopic model. Notably, WWOX significantly suppressed tumor growth (Figure 5a) and decreased tumor size (Figure 5b). Tumors from the sham-treated group grew 1.8 times larger than those expressing LV-WWOX and 5.2 times larger than those expressing H-LV-WWOX (*p* < 0.05). Using one-way ANOVA, the results of the F-test indicated a significant difference among the six groups, with a *p*-value of 0.002, which is less than 0.05. In the *sham-treated tumors* group, bladder tumor invasion extended from the urothelium to the muscle layer and was classified as stage T2. Additionally, the sham-treated tumor cells displayed increased variation in nuclear size and a higher frequency of mitotic figures. The LV group exhibited histological features characteristic of bladder tumors, including pronounced nuclear pleomorphism. In contrast, the H-LV-WWOX group displayed mild dysplasia and a high concentration of inflammatory cells, which inhibited tumor progression to an advanced stage (T1). Several tumor nests with localized cell debris further emphasized the anti-tumor activity of H-LV-WWOX in the bladder (Figure 5c). *t*-test analysis revealed no significant differences in body weight among the six groups on the day before treatment (0 d) (Figure 5d). However, 60 days post-treatment, the body weights of the animals in the H-LV group were lower than those of the animals in the sham-treated (tumor) group. Conversely, the H-LV-WWOX group showed a greater increase in body weight than the other groups did. The LV-WWOX and H-LV-WWOX groups exhibited no noticeable change in body weight, suggesting that H-LV-WWOX does not cause detrimental systemic toxicity in rats.

### 3.6. WWOX Increases TNF-α-Induced Caspase-3 Activation in the F344/AY-27 Rat Model

**Figure 6:**
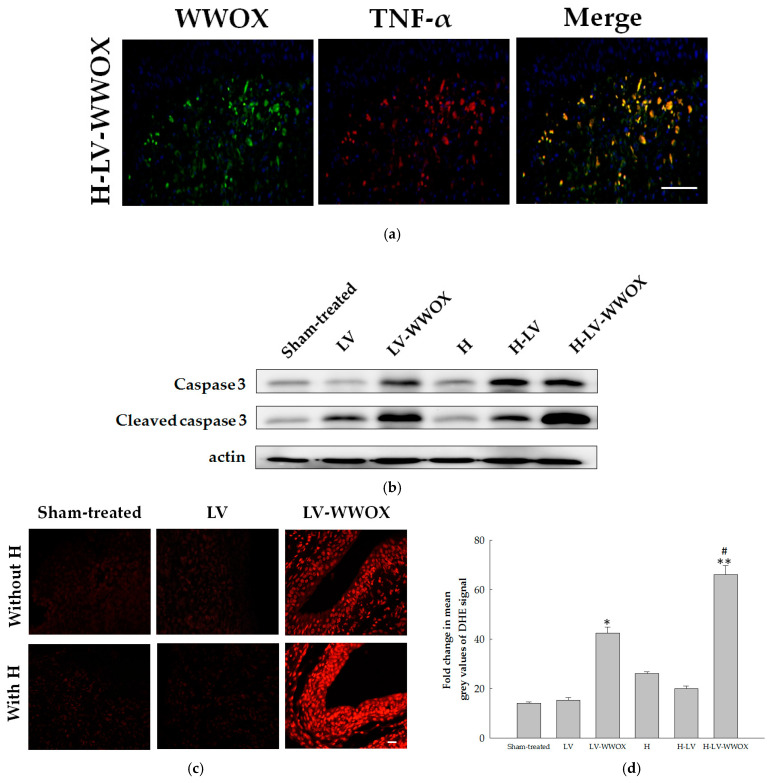
WWOX increases TNF-α-induced caspase-3 activation in the F344/AY-27 rat model. (**a**) Double-label immunofluorescence analysis of WWOX (green) and TNF-α (red) in F344/AY-27 rats treated with H-LV-WWOX. Scale bar = 100 μm. (**b**) The F344/AY-27 rat model was treated with LV, LV-WWOX, H, H-LV, and H-LV-WWOX, respectively. The expression levels of WWOX, caspase-3, and cleaved caspase-3 were assessed using western blotting, with actin serving as the loading control. (**c**) Representative pictures for DHE staining. (**d**) Quantification of DHE fluorescence by measuring the mean grey value of DHE signal using image j software. Data are presented as mean fold change compared to control ± SEM of five independent experiments. * *p* < 0.05 for sham-treated tumors versus LV-WWOX; ** *p* < 0.01 for sham-treated tumors versus H-LV-WWOX. # *p* < 0.05 for LV-WWOX versus H-LV-WWOX (magnification of ×400).

To further explore the mechanism underlying the antitumor effects of WWOX, specifically whether these effects can be attributed to TNF-α-mediated cell death, we investigated the expression and localization of TNF-α in the F344/AY-27 rat model treated with H-LV-WWOX. Both WWOX and TNF-α were expressed in rats treated with H-LV-WWOX. Immunofluorescent staining revealed the localization of WWOX and TNF-α, as shown in Figure 6a. These results suggest that overexpression of WWOX enhances the expression of TNF-α, with both proteins localizing to the cytoplasm. To determine whether the increased apoptosis induced by WWOX in the F344/AY-27 rat model treated with H-LV-WWOX is associated with enhanced TNF-α activation, we analyzed the kinetics of TNF-α and caspase-3 activation (Figure 6b). As shown in Figure 6b, the expression levels of caspase-3 protein in AY-27 cells infected with H-LV-WWOX were 1.16-fold higher (*p* < 0.01) compared to those infected with LV-WWOX. Additionally, the cleavage of caspase-3 protein expression levels in AY-27 cells infected with H-LV-WWOX were 1.22-fold higher (*p* < 0.01) than in the LV-WWOX group.

TNF-α may stimulate ROS production via several sources. We past investigated whether WWOX-induced TNF-α expression is due to activation of ROS generation. Here, we found that WWOX markedly induced superoxide and hydrogen peroxide production, determined by using DHE under a fluorescence microscope (Figure 6c). To test whether ROS are regulated by WWOX, we measured the ROS levels in response to F344/AY-27 rat model treated with H-LV-WWOX. ROS expression levels in H-LV-WWOX group were 4.68-fold higher (*p* < 0.01) than in the sham-treated group. ROS expression levels in LV-WWOX group were 3.01-fold higher (*p* < 0.05) than in the sham-treated group. ROS expression levels in H-LV-WWOX group were 1.56-fold higher (*p* < 0.01) than in the LV-WWOX group. These findings suggest that WWOX-induced TNF-α upregulation may suppress bladder tumorigenesis by promoting cellular death.

## 4. Discussion

This study emphasizes the successful utilization of modified gelatin-based nanocomposites as efficient platforms for localized and sustained gene delivery to the transitional epithelium via the intravesical route. A key finding was the ability of the hydrogel to encapsulate and protect the LV, allowing its sustained and controlled release. The porous structure of the hydrogel facilitated the gradual diffusion of viral particles, ensuring extended delivery of the LV. This sustained release is crucial for enhancing the transduction efficiency and achieving prolonged therapeutic effects, particularly in localized gene therapy [19]. A bioadhesive delivery system may overcome the limitation of drug retention time and enhance LV-mediated gene delivery while demonstrating minimal cytotoxicity and favorable interactions with host tissues [20].

This study successfully demonstrated that hydrogel-encapsulated LV-WWOX vectors can sustain the expression of WWOX protein in the urothelium. The effective release of the vector and the high transduction efficiency highlight the potential of H-LV-WWOX systems in improving gene therapy outcomes. This approach can result in more effective and targeted treatments by ensuring prolonged exposure and efficient delivery of therapeutic genes.

Intravesical delivery scaffolds provide efficient and highly localized delivery of therapeutic transgenes. Previous studies have demonstrated that the efficiency of scaffold-mediated transgene delivery depends on the robust and rapid infiltration of host cells, which can then physically encounter vectors and take up the transgenes [18]. The hydrogels prepared in this study demonstrated efficiency as scaffolds and reservoirs for lentiviruses that were resistant to urine voiding and enabled sustained gene expression. The LV-WWOX-hydrogel vector system offers a promising approach for localized gene delivery with sustained release and high transduction efficiency. In our discussion, Figure 3a illustrates the limitations of the current assay, including the low-level inflammation associated with the intravesical instillation of lentivirus. Since the inflammation caused by intravesical gene therapy is localized, and biochemical test results for all groups were within normal ranges (e.g., white blood cell count: Control/H-LV-WWOX was 7.3 ± 2.3 10^9^/L / 6.0 ± 1.4 ×10^9^/L), this indicates that bladder perfusion of H-LV-WWOX is unlikely to cause systemic side effects.

The relatively mild impact observed in the LV and LV-WWOX groups compared to that in the H-LV and H-LV-WWOX groups suggests that the hydrogel component may contribute to the inflammatory response. Further studies should focus on optimizing hydrogel formulations to minimize the adverse effects and enhance treatment responses. The CMG data support the idea that transurethral open CMG under saline infusion does not significantly alter bladder function or contribute to inflammatory responses. The observed differences in the histological outcomes and localized inflammation were likely specific to the treatment conditions rather than to the procedural process. This underscores the need for continued refinement of hydrogel-based delivery systems to minimize adverse effects while ensuring therapeutic efficacy. The intercontraction intervals, which reflect the bladder’s filling capacity and the mechanisms governing micturition, showed no significant differences between the experimental groups and the normal control (Figure 3c). This suggests that the infusion of LV-WWOX or H-LV-WWOX does not alter voiding cycles. Furthermore, the CMG data confirm that the hydrogel-based intravesical instillation of LV, LV-WWOX, H-LV, and H-LV-WWOX has no significant effect on bladder function. To evaluate the irritative effects of the drug on the bladder, this study assessed its impact on CMG via transurethral administration. Compared to the clinically used drug pharmorubicin, pharmorubicin exhibited significantly greater irritative effects than the H-LV-WWOX group. Histological analysis showed that H-LV-WWOX demonstrated superior inhibitory effects on bladder tumors compared to pharmorubicin and caused less irritation to the urothelium (Figure S1).

Furthermore, H-LV-WWOX strongly activated inflammatory responses and ROS generation in AY-27 bladder cancer cells. By influencing ROS levels, TNF-α enhances oxidative stress, which in turn can activate or amplify the WWOX pathway, leading to cell death [21]. This connection underscores the interplay between inflammatory signaling and oxidative stress in the regulation of WWOX-mediated apoptosis. WWOX overexpression led to significant morphological changes, increased ROS production, and marked inhibition of cell proliferation. Elevated caspase-3 activity in H-LV-WWOX-treated AY-27 cells highlights the role of WWOX in promoting apoptosis. Caspase-3, a key mediator of the apoptotic pathway, is crucial for inducing cell death, and its increased activity supports the proapoptotic effects of WWOX. This study’s findings suggest that WWOX enhances the apoptotic response to TNF-α by increasing caspase-3 activity, linking the TNF-α-ROS-caspase pathway to the observed antitumor effects. This mechanism is consistent with previous studies that have highlighted the role of WWOX in modulating apoptosis and its potential as a tumor suppressor [22].

Our previous studies have shown that WWOX strongly activates both the inflammatory response and ROS generation in the AY-27 bladder cancer cell line. ROS are recognized as key effector molecules in TNF-α-mediated cell death and can induce DNA damage and mutations. Overexpression of WWOX led to significant morphological changes, increased ROS production, and marked inhibition of cell proliferation [23]. To further explore the in vivo role of WWOX in cancer cell elimination, the F344/AY-27 rat orthotopic competition model was used. Previous studies have shown that WWOX induction triggers the production of various cytokines, such as TNF-α, IL-1, and IL-6 [24]. Immunohistochemical analysis revealed that the expression of both fluorescently tagged WWOX (green) and fluorescently labeled TNF-α (red) in F344/AY-27 rats resulted in cytosolic colocalization in over 80% of TNF-α/WWOX-expressing cells (Figure 6a). Moreover, no pathological changes were observed in the organs (heart, liver, lung, or kid6aney), suggesting that no systemic toxicities were induced by the lentivirus.

Taken together, this study advances our understanding of the ROS-induced anticancer effects of H-LV-WWOX in the following aspects: (i) the increase in ROS levels following WWOX overexpression leads to cellular oxidative stress, which promotes apoptosis by causing irreparable damage to cellular components and DNA, ultimately resulting in cell death; and (ii) H-LV-WWOX enhances the effects of TNF-α by promoting ROS production, leading to a more effective induction of cell death and improved antitumor activity. Understanding these mechanisms provides valuable insights into the therapeutic potential of H-LV-WWOX and supports its application in combination treatment strategies to enhance cancer treatment outcomes.

Hydrogel-mediated lentiviral gene delivery offers promising benefits, such as localized delivery, and reduced systemic toxicity. However, limitations include the possibility that the hydrogel may hinder viral diffusion in less permeable tissues, resulting in reduced transduction efficiency. Future advancements in material design and delivery methods could address these challenges.

## 5. Conclusions

This study demonstrates that H-LV-WWOX induces cytotoxicity and apoptosis in AY-27 bladder cancer cells. Additionally, TNF-α plays a significant role in the preventive effects against bladder cancer. WWOX enhances the pro-apoptotic effects of TNF-α by promoting ROS production. Furthermore, in an orthotopic bladder tumor model, H-LV-WWOX effectively inhibited tumor growth. These findings highlight the potential of H-LV-WWOX as a promising therapeutic strategy for bladder cancer, leveraging its ability to induce apoptosis and enhance the effects of TNF-α through ROS production.

## Data Availability

The raw data will be available from the corresponding author upon reasonable request.

## Supplementary Materials

The following supporting information can be downloaded at: https://www.mdpi.com/article/10.3390/pharmaceutics17020143/s1, Figure S1. Comparison of H-LV-WWOX Therapy with Current Clinical Medications. (a,b) Representative Continuous Cystometrogram (CMG) Recordings: Continuous CMG recordings were obtained from F344 rats during intravesical saline infusion (0.7 mL/min) under anesthesia. (c,d) Pathohistological Examination: F344 rats underwent intravesical instillation with either pharmorubicin or H-LV-WWOX treatments.

## References

[1] Kałuzińska Ż., Kołat D., Kośla K., Orzechowska M., Bednarek A.K., Płuciennik E. (2021). In vitro and in silico assessment of the effect of WWOX expression on invasiveness pathways associated with AP-2 transcription factors in bladder cancer. BMC Urol..

[2] Pospiech K., Płuciennik E., Bednarek A.K. (2018). WWOX Tumor Suppressor Gene in Breast Cancer, a Historical Perspective and Future Directions. Front. Oncol..

[3] Yang T., Xu R., Huo J., Wang B., Du X., Dai B., Zhu M., Zhan Y., Zhang D., Zhang Y. (2021). WWOX activation by toosendanin suppresses hepatocellular carcinoma metastasis through JAK2/Stat3 and Wnt/β-catenin signaling. Cancer Lett..

[4] Mo L., Li W., Shi X., Yang Z., Li X., Qin L., Luo Y., Mo W. (2017). WWOX suppresses proliferation and induces apoptosis via G2 arrest and caspase 3 pathway in nasopharyngeal carcinoma cells. Int. J. Clin. Exp. Pathol..

[5] Chen W., Zhou C., Zhang W., Atyah M., Yin Y., Guo L., Tang W., Dong Q., Ye Q., Ren N. (2018). Association of WWOX rs9926344 polymorphism with poor prognosis of hepatocellular carcinoma. J. Cancer.

[6] Tanna M., Aqeilan R.I. (2018). Modeling WWOX Loss of Function In Vivo: What Have We Learned?. Front. Oncol..

[7] Chen S.J., Huang S.S., Chang N.S. (2013). Role of WWOX and NF-κB in lung cancer progression. Transl. Respir. Med..

[8] Tsai C.W., Lai F.J., Sheu H.M., Lin Y.S., Chang T.H., Jan M.S., Chen S.M., Hsu P.C., Huang T.T., Huang T.C. (2013). WWOX suppresses autophagy for inducing apoptosis in methotrexate-treated human squamous cell carcinoma. Cell Death Dis..

[9] Lo J.Y., Chou Y.T., Lai F.J., Hsu L.J. (2015). Regulation of cell signaling and apoptosis by tumor suppressor WWOX. Exp. Biol. Med..

[10] Satapathy M.K., Nyambat B., Chiang C.W., Chen C.H., Wong P.C., Ho P.H., Jheng P.R., Burnouf T., Tseng C.L., Chuang E.Y. (2018). A Gelatin Hydrogel-Containing Nano-Organic PEI–Ppy with a Photothermal Responsive Effect for Tissue Engineering Applications. Molecules.

[11] Zöller K., To D., Bernkop-Schnürch A. (2025). Biomedical applications of functional hydrogels: Innovative developments, relevant clinical trials and advanced products. Biomaterials.

[12] Andreazza R., Morales A., Pieniz S., Labidi J. (2023). Gelatin-Based Hydrogels: Potential Biomaterials for Remediation. Polymers.

[13] Enayati M., Liu W., Madry H., Neisiany R.E., Cucchiarini M. (2024). Functionalized hydrogels as smart gene delivery systems to treat musculoskeletal disorders. Adv. Colloid. Interface Sci..

[14] Seidlits S.K., Gower R.M., Shepard J.A., Shea L.D. (2013). Hydrogels for lentiviral gene delivery. Expert Opin. Drug Deliv..

[15] McMahon S.S., Nikolskaya N., Choileáin S.N., Hennessy N., O’Brien T., Strappe P.M., Gorelov A., Rochev Y. (2011). Thermosensitive hydrogel for prolonged delivery of lentiviral vector expressing neurotrophin-3 in vitro. J. Gene Med..

[16] Wang X., Li C., Wang Y., Chen H., Zhang X., Luo C., Zhou W., Li L., Teng L., Yu H. (2022). Smart drug delivery systems for precise cancer therapy. Acta Pharm. Sin. B.

[17] Zhong R., Talebian S., Mendes B.B., Wallace G., Langer R., Conde J., Shi J. (2023). Hydrogels for RNA delivery. Nat. Mater..

[18] Liu C.W., Chang L.C., Lin K.J., Yu T.J., Tsai C.C., Wang H.K., Tsai T.R. (2014). Preparation and characterization of gelatin-based mucoadhesive nanocomposites as intravesical gene delivery scaffolds. Biomed. Res. Int..

[19] Sarfraz M., Qamar S., Rehman M.U., Tahir M.A., Ijaz M., Ahsan A., Asim M.H., Nazir I. (2022). Nano-Formulation Based Intravesical Drug Delivery Systems: An Overview of Versatile Approaches to Improve Urinary Bladder Diseases. Pharmaceutics.

[20] Yu L., Luo Z., Chen T., Ouyang Y., Xiao L., Liang S., Peng Z., Liu Y., Deng Y. (2022). Bioadhesive Nanoparticles for Local Drug Delivery. Int. J. Mol. Sci..

[21] Ju S., Singh M.K., Han S., Ranbhise J., Ha J., Choe W., Yoon K.S., Yeo S.G., Kim S.S., Kang I. (2024). Oxidative Stress and Cancer Therapy: Controlling Cancer Cells Using Reactive Oxygen Species. Int. J. Mol. Sci..

[22] Chen Y., Luo X., Xiao Z., Guo J., Cui Z. (2017). Exogenous WWOX enhances apoptosis and weakens metastasis in CNE2 nasopharyngeal carcinoma cells through the intrinsic apoptotic pathway. Int. J. Clin. Exp. Pathol..

[23] Liu C.W., Chen P.H., Yu T.J., Lin K.J., Chang L.C. (2022). WWOX Modulates ROS-Dependent Senescence in Bladder Cancer. Molecules.

[24] Shin M.J., Kim H.S., Lee P., Yang N.G., Kim J.Y., Eun Y.S., Lee W., Kim D., Lee Y., Jung K.E. (2023). Mechanistic Investigation of WWOX Function in NF-kB-Induced Skin Inflammation in Psoriasis. Int. J. Mol. Sci..

