# Supplementary material for: Novel Hydrogel-Mediated Lentiviral Gene Delivery via Intravesical Administration for Bladder Cancer Treatment
Source: Pharmaceutics. 2025 Jan 21;17(2):143. doi: 10.3390/pharmaceutics17020143 (PMC11858974; doi:10.3390/pharmaceutics17020143)
Supplement: Supplementary file 1 [file pharmaceutics-17-00143-s001.zip › pharmaceutics-3390755-supplementary.pdf]

Supplement data:

To evaluate the irritative effects of the drug on the bladder, this study assessed its impact on CMG via transurethral administration. Compared to the clinically used drug pharmorubicin (Figure S1a), pharmorubicin exhibited significantly higher irritative effects than the H-LV-WWOX group (Figure S1b). Histological analysis revealed that H-LV-WWOX (Figure S1d) demonstrated superior inhibitory effects on bladder tumors compared to pharmorubicin (Figure S1c) and caused less irritation to the urothelium.

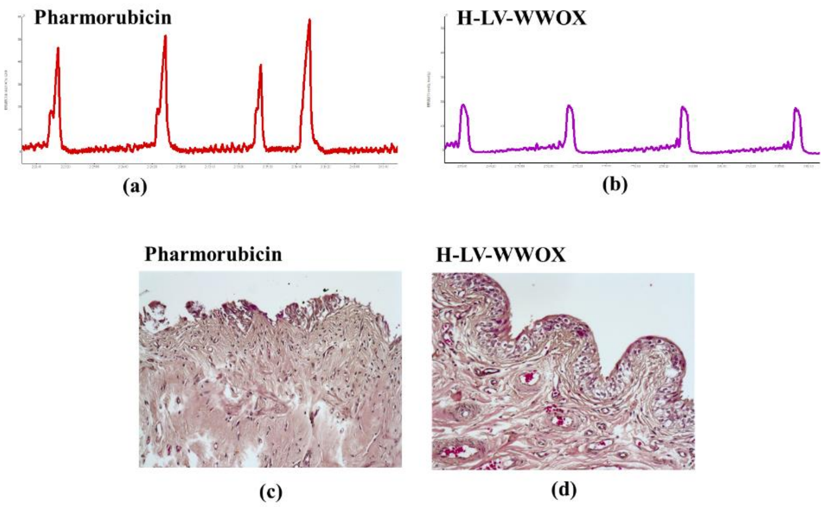

Supplementary Figure S1. Comparison of H-LV-WWOX Therapy with Current Clinical Medications. (a, b) Representative Continuous Cystometrogram (CMG) Recordings: Continuous CMG recordings were obtained from F344 rats during intravesical saline infusion (0.7 mL/min) under anesthesia. (c, d) Pathohistological Examination: F344 rats underwent intravesical instillation with either pharmorubicin or H-LV-WWOX treatments.
